# Retention of prions in the polychaete *Hediste diversicolor* and black soldier fly, *Hermetia illucens*, larvae after short-term experimental immersion and feeding with brain homogenate from scrapie infected sheep

**DOI:** 10.1016/j.heliyon.2024.e34848

**Published:** 2024-07-20

**Authors:** Sylvie L. Benestad, Linh Tran, Arne M. Malzahn, Nina S. Liland, Ikram Belghit, Andreas Hagemann

**Affiliations:** a Norwegian Veterinary Institute, P.O. Box 64, 1431, Ås, Norway; b SINTEF Ocean, Department of Fisheries and New Biomarine Industry, Brattørkaia 17C, 7010, Trondheim, Norway; c Institute of Marine Ecosystem and Fishery Science, University of Hamburg, 22767, Hamburg, Germany; d Institute of Marine Research, P.O. Box 1870, Nordnes, 5817, Bergen, Norway

**Keywords:** Fish sludge, Circular bioeconomy, Biosecurity, Feed safety regulation, Aquaculture

## Abstract

Finding alternative protein and lipid sources for aquafeeds is crucial for the sustainable growth of fed aquaculture. Upcycling industrial side streams and byproducts using extractive species can reduce waste and help reduce the sector's dependence on fish meal and fish oils. Polychaete worms (*Hediste diversicolor*) and black soldier fly (*Hermetia illucens*) larvae (BSFL) are promising candidates for converting waste materials into valuable protein and lipid sources. However, further research and evaluations are needed to ensure the safety and regulatory compliance of these alternative feed sources, especially regarding prions spreading potential in the unlikely case that prions would be introduced in the value chain via feedstocks. In the present investigation, BSFL and juvenile polychaetes that had received a massive dose of scrapie prions through immersion and oral inoculation were found to harbour detectable prions using an ultrasensitive amplification method known as PMCA. This observation suggests that both *H. diversicolor* and BSFL have the potential to serve as mechanical vectors for prions diseases. However, it is important to note that insects, lacking the prion protein gene, are incapable of propagating prions. Therefore, the quantity of prions present in the larvae will inevitably be lower than the amount of prions they encountered. This is the first study to report on the fate of prions through ingestion by these marine and terrestrial invertebrate species.

## Highlights

• Polychaetes and black soldier fly larvae (BSFL) were fed scrapie prions.
• Residual prions detected using a highly sensitive prion amplification method (PMCA).
• Polychaetes and BSFL can potentially serve as mechanical vectors for prion diseases.
• Scientifically implausible to consider them as reservoirs for prion replication.

## Graphical abstract

**Figure:**
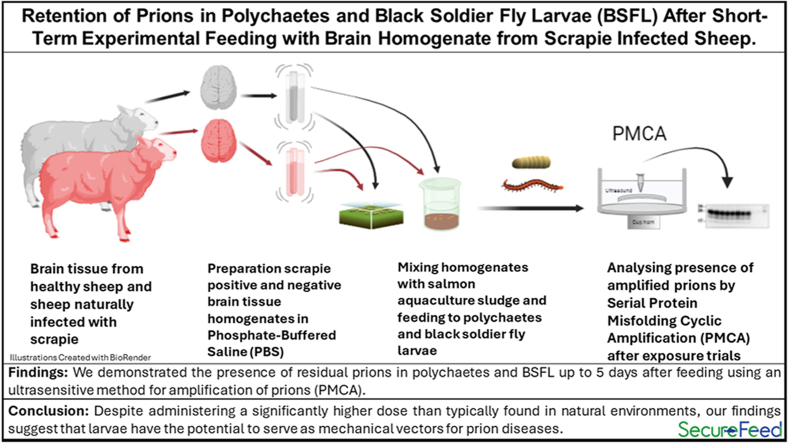

## 1 Introduction

In order for aquaculture to continue its sustainable growth and meet the demands of a growing world population, it must reduce its reliance on fish meal and fish oils. Over the past two decades, there has been a gradual shift towards using more plant- and animal byproduct-based aquafeeds, which has resulted in a decrease in the use of marine raw materials from capture fisheries [1,2]. However, this shift has led to competition between the aquaculture sector and terrestrial livestock production for resources [3,4]. There is an urgent need to find new, sustainable protein and lipid sources for aquafeeds which are not in direct competition with existing food production value chains and human nutrition. One solution lies in utilizing industrial sidestreams, wastes, and byproducts from both the blue and green food sectors to produce biomass of low-trophic extractive species. These resources can play a key role in the sustainable expansion of the fed aquaculture and aquafeed industries.

For example, the Norwegian Atlantic salmon farming industry produced approximately 0.5 million tons of solid waste in the form of salmon sludge in 2016 [5]. This waste, which consists of faeces and feed spill, can be repurposed to produce high-value proteins and lipids for animal feed. Wang et al. [6] estimated that as much as 70 % of the nutrients contained in aquafeeds may end up in the environment as feed spillage, faeces, respiration and excretion. While the dissolved nutrients could be utilized for production of “single-cell protein” (e.g. bacteria, microalgae) or macroalgae, extractive species such as polychaete worms (*Hediste diversicolor*) and black soldier fly (*Hermetia illucens*) larvae (BSFL) are excellent candidates for the upcycling of fish sludge to high value proteins and lipids for animal feed [[7], [8], [9], [10]]. Unlike other protein sources, the production of insect- or polychaete-based biomass, using fish sludge as feed, does not compete with human food sources. Black soldier fly larvae, for instance, are primarily used as a protein source to replace marine and plant-based ingredients in aquafeeds [[11], [12], [13]]. However, the lipid content consists mostly of saturated a fatty acids (58–72 %) and lack long-chained polyunsaturated fatty (LC-PUFA) acids such as eicosapentaenoic acid (EPA) and docosahexaenoic acid (DHA), required in the diet of carnivorous fish species [14]. Polychaetes, on the other hand, are rich in both marine proteins and fatty acids, making them a suitable substitute for both protein and lipid sources [7,15,16].

Processed animal protein (PAP) from seven different insect species, amongst them *H.* *illucens,* have been approved for use in aquafeeds by the European Union [EU No 2017/893; [17] in 2017 [18,18] under the prerequisite that they are reared on feed-grade substrate [2,19]. In 2021, silkworm (*Bombyx mori*) was included in the list of allowed insects along with an authorization to use insect PAP in feeds for pigs and poultry [EU No 2021/1372; 20]. However, regulations currently prohibit the use of salmon sludge as feedstock for production animals; *i*) Annex III to Regulation EC No 767/2009 [20] prohibits the use of urine, stomach- and gut content as feedstock for production animals, and *ii*) only animal byproducts classified as Category 3 are approved as feed for production animals and pets [EC No. 1069/2009 art. 14; [21]. To make this possible in the future, salmon sludge would need to be included in the animal by-product legislation [EC No 1069/2009 art. 14; [21], be classified as Category 3 material by the EU and undergo further research and scientific evaluation to ensure its safety [22]. While there is currently no push for regulatory changes in polychaete aquaculture in the circular economy, it is possible that this may change in the future as more evidence emerges about the nutritional content and bioremediation potential of polychaetes.

It is important to note that insects are considered farmed animals in the European Union and are subject to the same rules and regulations, including those aimed at preventing transmissible spongiform encephalopathies (TSEs) [EU No 2017/893; 17, EU No 2021/1372; 20]. TSEs or prion diseases are fatal neurodegenerative diseases that affect various species. The foremost examples are scrapie in sheep and goats, bovine spongiform encephalopathy (BSE) in cattle, chronic wasting disease (CWD) in cervids and sporadic Creutzfeldt-Jakob disease (sCJD) in humans. TSEs are due to an unconventional pathogen, namely the normal host encoded prion protein (PrP^c^), which undergoes a self-reproducing process and misfolds into conformers that are partially proteinase resistant (PrP^Sc^). These misfolded proteins, known as PrP^Sc^, or "prions" as short name for “**pro**teinaceous **in**fectious particle", accumulate in the central nervous system of infected individuals and eventually lead to cell death [23,24].

Prion diseases have long incubation periods (months or years), lack of specific immune response, and have currently no cure. They can occur sporadically/spontaneously (without known origin, mainly in old individuals), be genetic/inherited, or acquired, through exposure to or ingestion of infectious PrP^Sc^.

Knockout mice and goats with inherited lack of PrP^c^ cannot develop prion diseases [25,26] demonstrating that susceptibility to these illnesses depends on the presence of PrP^c^. Insect species have not been reported to possess a PrP homologue [27,28]. Since prion replication only occurs in hosts that express PrP^C^ [26,29,30], insects should not be able to replicate prions. However, some studies have suggested that insects could potentially act as mechanical vector for prion diseases [31,32]. However, the research on this topic in polychaetes is limited.

The diagnosis of prion diseases relies on the detection of PrP^Sc^, as it is the only reliable marker for infection. This is achieved using ELISA immunoassay or immunohistochemistry, and positive results are confirmed by western blotting, on post-mortem collected brain tissues [33,34]. These tools are considered gold standards and are used in large-scale sample analyses as part of TSE surveillance programs implemented in Europe. These programs were established in response to the major outbreak of BSE in the United Kingdom in 1986, which resulted in 180,000 diagnosed cases in cattle [33,34], and the recognition, ten years later, that BSE was causing a new human TSE (variant Creutzfeldt-Jakob disease, vCJD) [35]. This unprecedented public health crisis led to the adoption of the EU Regulation No 999/2001 [36] which set out rules to prevent, control and eradicate transmissible spongiform encephalopathies (TSEs) in Europe.

Put in place in Europe in early 2000 and still followed nowadays, surveillance programs have given a useful overview about the updated frequency of prion diseases in European small ruminants and cattle populations and have demonstrated the effectiveness of disease control measures. The measures implemented to combat prion diseases, such as banning the use of mammalian Meat and Bone Meal in livestock feed and avoiding the recycling of specified risk materials (SRMs) [37], have significantly reduced the occurrence of classical BSE and classical scrapie [37]. Classical BSE, demonstrated as being due to the consumption of contaminated Meat and Bone Meal, is now nearly eradicated. Classical scrapie, which is highly contagious between sheep, also appears to be under control with the majority of cases (91.7 %) reported in 2019 originating from four countries [38].

The surveillance has also led to the identification of novel prion strains, referred to as atypical, in sheep and goats (termed Nor98 or atypical scrapie) [39,40] and in cattle, (termed l-BSE and H-BSE) [41,42]. The usual type of the disease has been since named “classical” (Classical BSE abbreviated C-BSE), and Classical scrapie. While C-BSE occurs through the consumption of contaminated feed, the atypical cases in sheep and cattle occur sporadically and evenly across populations and are believed to arise spontaneously particularly in older animals. These atypical cases are therefore likely to persist and present an ongoing risk for the re-introduction and re-circulation of prion diseases into animal populations. As a result, the scientific community recommends the continued implementation of disease control measures and effective disease surveillance [37].

In Europe, the occurrence of scrapie in sheep has been described 250 years ago while BSE in cattle was first identified 40 years ago in the United Kingdom. Chronic wasting disease (CWD), the prion disease that affects cervids, was discovered in 1967 in Colorado, USA, and has since inexorably expended to 34 American states and 4 Canadian provinces. In April 2016, CWD was detected for the first time in Europe, specifically in a Norwegian reindeer, followed by two Norwegian moose two months later [43,44]. A subsequent surveillance programme for CWD was launched in the seven European countries with reindeer and moose populations, and consequently, 43 CWD cases are today detected in Europe, 37 in Norway including reindeer, moose and red deer, and three cases in Finland and four in Sweden, all in moose.

Despite the fact that CWD is caused by a misfolded host protein without genetic material, it is well-known that there are different strains of prions, which can influence the incubation period, disease phenotype, pattern of PrP^Sc^ deposition in the brain, biochemical characteristics of PrP^Sc^ and importantly the presence or absence of PrP^Sc^ in the peripheral tissues. These factors also influence the ability of an individual to shed infectivity, contributing to the potential for horizontal transmission between living animals. The differences of strains are attributed to distinct structural conformations of the PrP^Sc^ isoforms [45,46].

Prions are highly resilient to most disinfection and sterilisation procedures, such as heat, formalin, and irradiation [47,48]. This emphasizes the importance of preventing the entry and spread of prions in food, feed and environment to minimize the recycling of prions.

In the past two decades, more sensitive detection methods, called amplification methods, have been developed. One such technique is the Protein Misfolding Cyclic Amplification (PMCA) assay. This in vitro technique mimics, in an accelerated manner, the misfolding chain reaction that occurs in vivo, inducing the replication of PrP^Sc^ by recruiting normal PrP^c^ [49,50]. The objective is to amplify PrP^Sc^, also known as seeds, that may be present at very low levels in tissues making them undetectable using conventional diagnostics methods. This PMCA process involves cycles of sonication and incubation, which exponentially increase the amount of PrP^Sc^. After amplification, PrP^Sc^ is detected and visualized through Western blot following digestion of PrP^c^ with proteinase K.

Transmission of prion diseases is relatively easy between individuals of the same species through intracerebral injection of brain tissue from infected animals. However, transmission between different species is limited by a phenomenon called species barrier, often resulting in a lack of transmission or a low or inconsistent transmission. While factors such as compatibility between the primary amino acid sequence of the donor and the host PrP, the dose of infectious material, and the strain of prions seem to be essential, the interspecies propagation process remain largely unpredictable [51].

A prerequisite for utilizing household wastes and fish sludge to produce biomass of low-trophic extractive species, is that it is safe for both the production animal and the consumers. This study aims to assess, for the first time, whether *H.* *diversicolor* and *H.* *illucens*, two invertebrates which can contribute significantly to the circular bioeconomy if allowed to up-cycle such sidestreams, can serve as potential vectors for prion diseases. Juvenile polychaetes and BSFL were exposed with prions from brain homogenates of scrapie-positive sheep mixed or not with aquaculture sludge.

## 2 Materials and methods

### 2.1 Animals and husbandry

The polychaete *H.* *diversicolor* (OF Müller, 1776) (Annelida: Nereidae) used for this experiment were collected from intertidal mudflats in Trondheim Fjord, in Leangbukta, Norway [52]. The collected polychaetes were immediately transported to a holding tank in the laboratories at SINTEF SeaLab, Trondheim, Norway, and acclimatized for one week in flow-through holding tanks. The tanks, measuring 52x36 × 18 cm, contained natural sediment collected on site with sufficient water flow (20 μm sand filtered, 1 μm bag filtered seawater holding 10 °C, 35 ‰ salinity, 7.6 pH) collected at 70 m depth from the Trondheim Fjord) at a 16L:8D photoperiod. During the acclimatization period, the polychaetes were fed commercial fish feed (GEMMA DIAMOND 96 1.0, Skretting AS, Norway). Prior to transportation to the experimental site, live and healthy immature individuals were carefully selected and placed in new sediment (sand) one week in advance.

The larvae of black soldier fly (*H.* *illucens*) (BSFL, 7 days post hatch) used in this experiment were shipped from Protix Biosystems BV (The Netherlands) to the Institute of Marine Research (IMR) in Bergen (import reference No. 23SD48BB). Upon arrival, the larvae were repacked in clear plastic containers with perforated lids and sent to the Veterinary Institute in Ås, Norway, using express courier service over night.

### 2.2 Prion inoculum preparation

To feed both the insects and polychaetes, we used brain tissue homogenates which were prepared from sheep naturally infected with scrapie or from scrapie-negative sheep. The homogenates were diluted at concentrations of 40 and 20 % in Phosphate-Buffered Saline (PBS) for positive and negative samples, respectively. Scrapie positive brain tissues were secondary samples kindly provided by Dr. Andréoletti at the INRAe Toulouse and consisted of a pool of brain tissues from three different animals. These three scrapie positive donor sheep originated from the Langlade flock at INRA Toulouse. They were naturally affected with classical scrapie and were carrying the same PrP genotype V_136_R_154_Q_171_/V_136_R_154_Q_17_ as the sheep brain used as substrate for the PMCA analysis. The positivity of the tissues was confirmed at their arrival at the Norwegian Veterinary Institute by ELISA (HerdChek from IDEXX) and Western blot (TeSeE WESTERN BLOT from Bio-Rad). The negative brain tissues were secondary samples collected by veterinarians at the slaughterhouses as part of the European scrapie surveillance programme sent to the Norwegian Veterinary Institute laboratory for routine scrapie examination, tested negative with ELISA (HerdChek from IDEXX).

The salmon sludge used to feed polychaetes and BSFL comprised a mixture of sludges obtained from thirteen different smolt production facilities in Norway. Equal aliquots of the sludges were mixed with a stand mixer to create a homogenous solution of approximately 30 % dry matter, which has been found to be optimal for insects [9].

### 2.3 Polychaete experimental set-up

The day prior to transporting the worms to the Veterinary Institute in Ås, Norway, National Reference Laboratory for TSE and WOAH Reference Laboratory for CWD, polychaetes were allowed to evacuate their guts in cups containing clean seawater without any sediment for a minimum of 4 h. This duration has been proven effective in achieving empty guts in *H. diversicolor* [7]. Groups of ten polychaetes were subsequently weighed on a balance (Science Education, SE622, VWR, Italy) in a tared beaker containing tempered seawater and stocked into pre-labelled glass beakers. The combined wet weight of the polychaetes in each replicate was used to calculate daily feed rations based on the estimated nitrogen content of the diets and the polychaetes, following the method described by Malzahn et al. [8]. The glass beakers, measuring 1-L with a diameter of 95 mm, were filled with approximately 200 mL of sand, resulting in an approximately 8 cm thick sediment layer.

On the day of transport to Ås, most of the seawater above the sediment was removed prior to placing the beakers with polychaetes (10 ind. per beaker) in cooling boxes (24-L, Biltema, Art. 49–513, Norway) set to 4 °C. At the Norwegian Veterinary Institute, the worms were placed in a wine cooler cabinet (Cavecool Retro Obsidian, Hillerød, Denmark), located in the BSL-3 facilities dedicated to prions. This ensured a suitable temperature of 10–11 °C throughout the experiment.

**Fig. 1:**
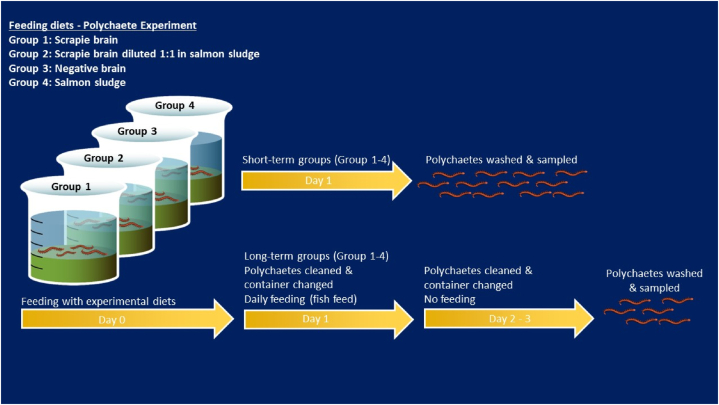
Flow-chart for the experimental set-up of the polychaete experiment exposing *Hedistediversicolor* to brain homogenates from sheep naturally infected with classical scrapie in short- and long-term.

The experimental set up is depicted in Fig. 1. The polychaetes which were divided in four groups (each one in triplicates, each in a beaker) were fed per beaker: 1) 800 μl of 40 % homogenate from sheep infected with classical scrapie, 2) 400 μl of 20 % homogenate from sheep naturally infected with classical scrapie mixed with 0.4 g of salmon sludge, 3) 1600 μl of 20 % homogenate (corresponding to 800 μl of 40 % prion positive homogenate given to Group 1) from sheep negative for scrapie, or 4) 0.8 g of salmon sludge. Each group underwent both short-term (1 day) and long-term (3 days) experiments. For the short-term groups, used as positive (Group 1 and 2) and negative controls (Group 3 and 4) treatment, the polychaetes were fed for one day before being extracted from the sediment, rinsed in fresh seawater to remove any debris and mucus, gently dried, and sacrificed by freezing at −20 °C before analyses. The ingested material remained in their digestive tracts at this stage. In the long-term experiment, the worms were extracted from the sediment after one day of feeding, rinsed and transferred to new beakers with clean sediment and seawater where they were allowed to evacuate their gut in a new beaker with clean sediment and seawater, and fed fish feed (GEMMA DIAMOND 96 1.0, Skretting AS, Norway) for one day. This process was repeated the following day, excluding the provision of feed. On the third day of the experiment, the polychaetes were extracted from the sediment, rinsed in fresh seawater, gently dried and weighed, and sacrificed by freezing at −20 °C before analyses.

### 2.4 Black soldier fly larvae experimental set-up

The experiment was initiated with approximately 150 BSFL (50 mg wet weight per individual) per treatment and four different treatments, divided into eight plastic boxes. Each group was fed similarly as with the polychaetes, with the exception of Group 2 which received the same amount of prions as Group 1 but mixed with salmon sludge. The treatments were as follows: 1) 2 ml of 40 % scrapie brain homogenate, 2) 2 ml of 40 % scrapie brain homogenate mixed with 2 g of salmon sludge, 3) 4 ml of 20 % negative brain homogenate, and 4) 4 ml of salmon sludge.

**Fig. 2:**
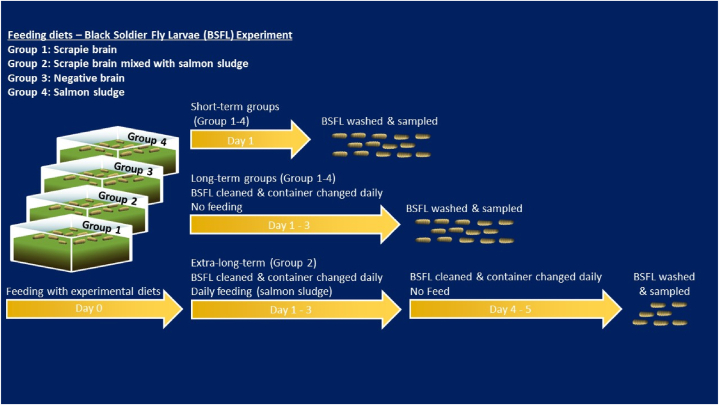
Flow-chart for the experimental set-up of the black soldier fly (*Hermetia illucens*) larvae (BSFL) experiment exposing *H. illucens* to brain homogenates from sheep naturally infected with classical scrapie in short-, long- and extra-long-term.

The experimental set-up is illustrated in Fig. 2. Each treatment underwent both short-term (1 day, 50 larvae per sample) and long-term (3 days, 100 larvae per sample) exposure experiments, apart from Group 2, which had an additional long-term treatment duration (5 days, 50 larvae per sample). The larvae were placed on a heating mat 56W (117 x 27,5 cm, Exotic life, The Netherlands) with thermostats 300W (Model PT2457, Exoterra/Hagen group, Montreal, Canada) set to 35 °C immediately upon arrival in the BSL3 facilities dedicated to prions. The larvae in the short-term, positive control treatment were sampled after one day when they had ingested all the provided feed. The larvae were carefully picked from the box using tweezers, transferred to strainers, cleaned in lukewarm water five times, dried with paper tissue, and then frozen at −20 °C. The larvae in the long-term treatments were also rinsed after one day, transferred into new plastic containers daily, and fed with salmon sludge for two days. The larvae were then sampled using the same procedure as the short-term treatment and frozen. An additional extra-long-term group was established with the same feeding and treatment regime as Group 2, but the larvae were left for an extra day without food in a clean box before being washed and sacrificed.

### 2.5 Analyses

200 mg of polychaetes or BSFL were transferred into grinding tubes containing 1 ml PBS and ceramic beads. The samples were then ground using a ribolyser (Precess 48, Bio-Rad), for two rounds of 20 s each at 6500 rpm, making 20 % whole larvae homogenates that were stored at −20 °C until analysis.

#### 2.5.1 Enzyme-linked immunosorbent assay (ELISA)

For the ELISA analysis, the HerdChek BSE-Scrapie Ag Test Kit (IDEXX) was used to detect the abnormal prion protein (PrP^Sc^) following the manufacturer's recommended procedures. The conjugate indicated for scrapie prions was used. Briefly, 120 μl of homogenates were mixed with 30 μl of the working plate diluent solution. Then, 100 μl of the mixture were loaded onto the antigen-capture plate, which was gently shaken for 45 min (200 ± 100 rpm) at room temperature on a shaking plate. After washing followed a 10 min incubation with 100 μl of conditioning buffer and a 45-min incubation with 100 μl conjugate with horseradish peroxidase (HRPO). The color was revealed by adding 100 μl Tetramethylbenzidine for 15 min, and the sample absorbance was read using 450 and 620 nm filters. The cut off value was calculated from 0.120 plus the mean of the two negative kit controls. All OD values below the cut off values are considered negative.

#### 2.5.2 Western blotting on non-amplified samples

Western blot was performed on the samples using the commercially available kit TeSeE Western Blot from Bio-Rad. The manufacturer's recommendations were followed, as previously described [53]. Briefly, the PrP^c^ contained in the homogenates was digested by incubation with proteinase K (20 μl per ml) at 37 °C for 10 min. The separation of the protein fragments was then carried out via electrophoresis using mini-PROTEAN TGX Precasts. The proteins from the gels were transferred onto polyvinylidene fluoride (PVDF) membrane (Bio-Rad) using a semi-dry transfer apparatus (Trans-Blot® Turbo™ Midi PVDF Transfer Packs, Bio-Rad). Followed the immunodetection process of PrP^Sc^, carried out using monoclonal antibodies SHa31 (primary antibody from the kit raised against the core protein amino sequence 148YEDRYYRE155), and the secondary antibody conjugated with horseradish peroxidase.

The membrane was developed with SuperSignal West Pico Plus Chemiluminescent substrate (Thermo-Fisher) and the signals were visualized using Azure c250 (Azure biosystems, California, USA). The samples were considered positive when the characteristic banding patterns of PK-resistant core of PrP^Sc^ were visible.

#### 2.5.3 Protein Misfolding Cyclic Amplification (PMCA)

Due to the poor results obtained from ELISA and Western blot analyses (Supplementary Fig. 1) in detecting PrP^Sc^ in the worms, including those from the positive control groups, probably due to small amounts of prions ingested by the worms, it was decided to use the more sensitive assay PMCA. To amplify prion seeds from a sample, PMCA requires an amplification substrate, cellular prion protein (PrP^c^) obtained from normal brain homogenates of a susceptible host. Previously, we have found that brain homogenate from a scrapie sheep carrying the genotype V_136_R_154_Q_171/_ V_136_R_154_Q_171,_ which is highly susceptible to classical scrapie, was an efficient substrate to amplify scrapie seeds [54]. PMCA was performed as described in Harpaz et al. [54]. Briefly, the PMCA substrate (normal PrP^c^) consisted in a 10 % w/v homogenate of brains from healthy sheep (V_136_R_154_Q_171/_ V_136_R_154_Q_171_) obtained as secondary samples either during necropsies conducted at the Section for Small Ruminant Research and Herd Health, Faculty of Veterinary Medicine, Norwegian University of Life Sciences, or directly after slaughter at the Nortura Forus abattoir. The collected brains were immediately frozen at −70 °C following sampling as described in Harpaz et al. [54]. Homogenates were prepared in PMCA conversion buffer which contained 1X PBS, 1 % Triton X-100, 150 mM NaCl, and a protease inhibitor cocktail (Roche) at a ratio of 1/10. The 10 % homogenates of polychaetes or BSFL were centrifuged at 800 g at 4 °C for 2 min and the supernatant was transferred into Eppendorf tubes and frozen.

To initiate the PMCA procedure, the substrate was thawed and heparin (100 μg/ml) and digitonin (0.005 %) were added. Then, 90 μl of this PMCA substrate were mixed with 10 μl of 10 % polychaetes or BSF homogenate (final larvae dilution of 10^−2^) in a PCR tube containing two Teflon beads. The tube was placed in a Q700 sonicator (Qsonica) and submitted to 48-h cycles of incubation and sonication at 37 °C. Each PMCA cycle consisted in 29 min of incubation and 30 s of sonication at 150–170 W. A new round started after each 48-h cycle, with 10 μl of the amplification product adding to 90 μl of fresh substrate. Negative controls, consisting of homogenates from corresponding larvae that were not in contact with PrP^Sc^ (group 3 and 4) were included in each PMCA experiment to monitor for potential cross-contamination. Each sample underwent triplicate testing in three consecutive rounds of PMCA, utilizing the 10^−2^ dilution. To visualize the presence of PrP^Sc^ in the PMCA amplified products, Western blot was performed for each round and sample after digestion of PrP^c^ with Proteinase K. We did not perform any inhibition study of the insect's matrix for the PMCA method, however, the positive amplification in all sample groups suggests that there was small, if any, inhibition of the insect matrix. Each sample was analysed at least in triplicates, at one dilution (10^−2^), and for a duration of three rounds. A sample was considered positive when at least two of the three triplicates showed amplification products as visualized by Western blot.

#### 2.5.4 Western blotting on PMCA products

The Western Blot method (using TeSeE Western Blot from Bio-Rad) was employed to visualize PrP^Sc^ in the amplification PMCA products. The manufacturer's recommendations were followed, as described above, with slight modification for the digestion step of the normal cellular prion protein PrP^c^ with PK (Sigma-Aldrich). The PK concentration was increased to 100 μl/ml final concentration at 37 °C and the digestion was carried out for 90 min.

## 3 Results

To initiate the study, the ELISA techniques for detection of PrP^Sc^ was employed to test all groups of BSFL and polychaetes. Only the 20 % homogenate samples from BSFL Group 1 and 2 in the short-term experiment yielded positive results (Supplementary Table 1). Surprisingly, the positive control polychaetes from Group 1 and 2 in the short-term experiment displayed negative results (Supplementary Table 1). To assess whether the whole larvae homogenate could impact on the efficiency of the ELISA test, we compared the ELISA results after spiking PBS, polychaetes or BSFL tissues with serial dilutions of PrP^Sc^ from the same scrapie sheep brain homogenate as used for the exposure experiment homogenate. The results revealed that homogenates from worms, especially polychaetes interfered with the tests (Supplementary Fig. 1). In contrast, the Western blot detection process was not hindered by worm tissues, as demonstrated by the presence of PrP^Sc^ in both the larvae homogenates and PBS (see Supplementary Fig. 1).

These findings were not deemed satisfactory, prompting us to employ the more sensitive PMCA method to amplify the prions contained in the homogenates. Subsequently we examined various samples from each group, analyzing dilutions ranging from 10^−2^ to 10^−7^, to identify the optimal dilution for successful seed amplification (data not shown). The investigation revealed that the dilution with the highest level of effectiveness was 10^−2^, which was then used throughout the entire study to assess the presence or absence of PrP^Sc^ in the samples.

**Fig. 3:**
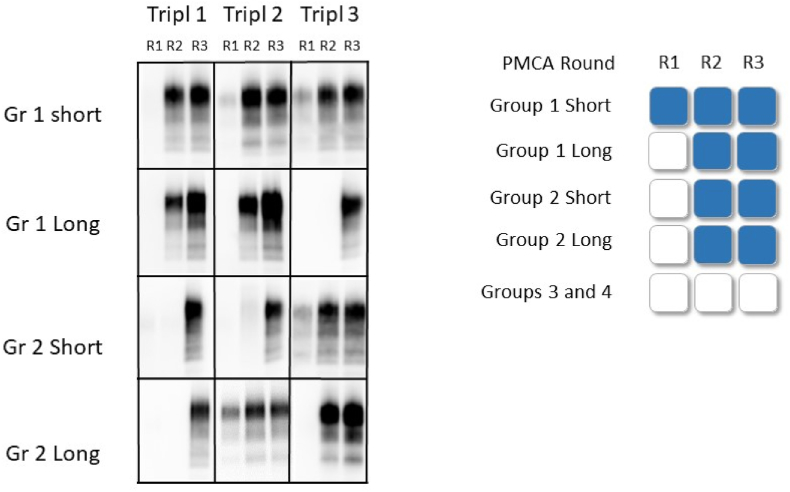
Polychaetes analysis: Left panel: The presence of PrP^Sc^ in the PMCA products was visualized by Western blot, from the same group in triplicates (T1, T2, T3). Group 1: Polychaetes larvae fed scrapie positive brain; Group 2: Larvae fed scrapie positive brain diluted 1:2 in salmon sludge; R1: after 1 round of PMCA, R2: after 2 rounds of PMCA; R3: after 3 rounds of PMCA. Short: The polychaetes were washed and sacrificed immediately after being fed 24 h with prions. Long: the polychaetes were allowed to empty their gut after one day feeding before being washed and sacrificed. Right panel: Schematic representation of the PMCA results for each group. The empty squares indicate no amplification, the blue squares indicate amplification of prions of at least 2 out of 3 (triplicate) samples. Group 3: Larvae were fed normal brain material (no prions); Group 4: larvae were fed salmon sludge.

**Fig. 4:**
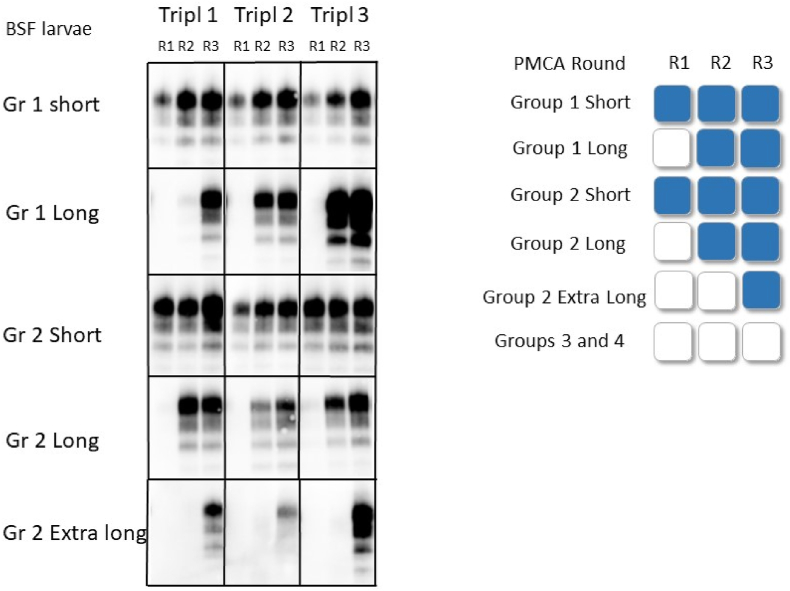
BSF larvae analysis: Left panel: The presence of PrP^Sc^ in the PMCA products was visualized by Western blot, from the same group in triplicates (T1, T2, T3). Group 1: larvae fed full scrapie positive brain; Group 2: larvae fed scrapie positive brain diluted 1:2 in sludge; Short protocol: larvae were washed and sacrificed immediately after being fed 24 h with prions; Long protocol: larvae were allowed to empty their gut after one day feeding before being washed and sacrificed; Extra-long protocol, the BSFL were fed three extra days with sludge before being washed and sacrificed; R1: after 1 round of PMCA, R2: after 2 rounds of PMCA; R3: after 3 rounds of PMCA. Right panel: Schematic representation of the PMCA results for each group. The empty squares indicate no amplification, the blue squares indicate amplification of prions of at least 2 out of 3 (triplicate) samples. Group 3: Larvae were fed normal brain material (no prions); Group 4: larvae were fed salmon sludge.

Remarkably, the PMCA results unveiled the presence of prions in each group of larvae (both polychaetes and BSF) that were immersed/fed with prions (Fig. 3, Fig. 4 and Supplementary Fig. 3). PrP^Sc^ was detected in the groups exposed to either a full positive brain (Group 1) or a positive brain diluted 1:2 with sludge (Group 2), regardless of whether they were immediately sacrificed after feeding (short-term), had emptied their gut over 3 days (long-term), or for the BSFL, allowed to live 4 days (extra-long) after feeding with prions.

## 4 Discussion

Using ultra-sensitive state-of-the art detection methods, we were able to uncover small amounts of residual prions in polychaetes and BSFL immersed in and fed with scrapie prion, even after purging the animals for a period long enough to cleanse their digestive systems [55].

There are conflicting results in the existing literature regarding the presence of PrP^Sc^ content after ingestion and digestion. Some *in-vitro* studies investigated the effects of digestive fluids of cattle and sheep on prions, with two of them showing a reduction of detectable prions after in vitro-digestion [56,57] while a third study did not show any reduction in similar condition with sheep digestion fluids [58].

Additionally, *in-vivo* experiments have detected PrP^Sc^ in faeces of different species fed with prions. VerCauteren et al., 2012, demonstrated infectivity of PrP^Sc^ in faeces from crows gavaged with prions [59]. Nichols et al. [60] showed that the digestive systems of coyotes do not eliminate infectious prions as their faeces still contained infectious prions for up to three days after ingesting homogenate from elk affected by CWD. Baune et al. [61] investigated the presence of prions in their faeces after feeding with meat spiked with CWD prions, and the quantification they performed indicated that the vast majority of prions (over 96 %) were eliminated during the first defecation.

In insects, other studies support our findings in different insects than BSFL or polychaetes, or in fish. For example, Wisniewski et al. [31] showed the presence of infectivity in hay mites found in herds with sheep affected by scrapie, and these results were confirmed by the same group by subsequent passages in mice [62]. This group postulated at this stage that mites could not only act as mechanical vector, but could also replicate prions, but no further studies could support this speculation. Post et al. [32] showed that PrP^Sc^ could be detected in the content of larvae and pupae from the *Sacrophaga carnaria* fed with scrapie prions, and that hamsters fed with these larvae could transmit the disease to hamsters by subsequent inoculation.

Conversely, attempts to transmit scrapie through various methods involving nematode parasites failed [63,64]. Overall, these experiments suggest that nematode parasites do not accumulate enough infectivity to transmit scrapie to sheep through larvae ingestion. Nevertheless, a more recent study detected prions by PMCA in earthworms exposed to prion-contaminated soil, and in some worms even after 28 days post exposure, suggesting earthworms could act as vectors for prion disease transmission [65]. More recently, Haley et al. [66] showed that CWD prions seeding activity was detected by RT-QUiC amplification method in one out of 30 ticks collected from elk in endemic CWD areas. This tick was collected from an individual at terminal stage of the disease. Additionally, prion seeding activity has been found in blacklegged ticks (*Ixodes scapularis*) experimentally fed brain sample from a late-stage CWD-positive white-tailed deer (*Odocoileus virginianus*) [67].

In the realm of fish, Ingrosso et al. [68] showed that fish challenged with a significant amount of scrapie mouse brain homogenate through oral or parenteral routes were able to clear the majority of infectivity load. When mice were intracerebrally inoculated with fish tissue collected at different time points after oral or parenteral inoculation, none of the mice develop prion disease. However, a small number of recipient mice did show the presence of PrP^Sc^ and spongiform lesions in the brain. This study also observed specific binding of PrP^Sc^ to the mucosal side of fish intestine in the absence of an active uptake of the prion protein through the intestinal wall suggesting that prion protein uptake through the intestinal wall may not be an active process. Unfortunately, our study in larvae could not determine whether prions are sequestered in the intestinal mucosa or if epithelial cells in the larvae actively uptake them.

Due to the limited sensitivity of the traditional techniques, we employed the ultrasensitive amplification method known as PMCA. It is important to note that the use of PMCA may lead to overestimation of results, as it is shown to be between 160 and 105 folds more sensitive than the bioassay in rodents, which serves as the gold standard for assessing prion infectivity potential [[69], [70], [71]]. Additionally, in our present study, the larvae were fed the maximum dosage of prions to investigate whether they would retain these prions as proof of evidence. However, this approach does not replicate a real-life scenario. Furthermore, as the aim of the present study was to assess the presence or absence of prions in the samples, no attempt was made to quantify the prions. Currently, it is unknown whether polychaetes, like insects, lack PrP^c^ homologues as indicated by Raeber et al. [27]. Nevertheless, no invertebrate, to our knowledge, has yet been found to possess PrP^c^, and BSFL and polychaetes may primarily act as mechanical vectors for prions acquired through their diets.

The risk of polychaetes, which serve as production animals upcycling e.g. aquaculture side streams, ingesting bovine material has been effectively minimized since 2001 through the ban on using such materials as feed, as stated in Regulation EC No. 999/2001 [36]. The necessity of a ban of bovine material from fish feeds was reconfirmed in 2004 when an EFSA opinion underlined the risk of water contamination during slaughtering, from blood and other tissues of BSE infected cattle [72]. In 2007, EFSA published another scientific opinion on the potential risk of spray-dried bovine blood as an ingredient in fish feed but was unable to draw definitive conclusions due to insufficient research data. It is worth noting that the inclusion of processed bovine blood products in the authorized ingredients list for fish feed would hinder the ability to detect prohibited bovine by-products, as emphasized by the authors. However, based on a subsequent scientific opinion by EFSA in 2020, the risks of inducing a new case of BSE in the cattle population using ruminant collagen and gelatine in fish feed were deemed minimal. Consequently, the European Commission amended Regulation EC No. 999/2001 in 2021 [73], permitting the use of gelatine and collagen from ruminants in feed for non-ruminant animals, including fish in aquaculture. This regulatory change represents the first step towards utilizing animal by-products from cattle for fish feed, based on the assessment that the residual BSE infectivity would be negligible given the current epidemiological situation of BSE and the implemented control measures within the EU.

## 5 Conclusion

The expression of the normal cellular prion protein (PrP) is lacking in insects and invertebrates [27]. Consequently, larvae are unable to replicate prions, resulting in a lower level of prions within their bodies compared to the dose they are in contact with or consume. However, this study found that *H. illucens* larvae and *H. diversicolor* exposed to and orally inoculated with a substantial dose of prions retained detectable levels of prions using the ultrasensitive amplification method (PMCA).

Currently, there are no ongoing bioassay studies to determine whether such small amounts of PrP^Sc^ remaining in the worms after digestion and defecation could be sufficient to cause the transmission of prion disease in e.g. mice models. Hence, the resulting risk of infecting susceptible animals with the amount of PrP^Sc^ contained in the invertebrates used in this study is unclear and needs further investigations.

## Data availability

The raw data supporting the conclusions of this manuscript will be made available by the authors to any qualified researchers upon request.

## Ethics statement

The scrapie positive brain samples provided by Dr Andréoletti at the INRAe Toulouse were secondary samples from scrapie positive sheep from naturally sick animals collected post-mortem. There is no ethical approval necessary for these samples in France. The negative sheep brain samples, used as negative controls, were collected by veterinarians at slaughterhouses as part of the National scrapie surveillance programme, organised and financed by the Norwegian Food Safety Authority, and sent to the Norwegian Veterinary Institute for TSE testing. The brain tissue from healthy sheep which was used as substrate for the PMCA were secondary samples collected at slaughterhouse by Dr Ersdal at the Norwegian University of Life Sciences. There is no ethical approval necessary for these samples in Norway.

## CRediT authorship contribution statement

**Sylvie L. Benestad:** Writing – review & editing, Writing – original draft, Visualization, Validation, Resources, Methodology, Investigation, Formal analysis, Data curation, Conceptualization. **Linh Tran:** Validation, Resources, Methodology, Investigation, Formal analysis, Data curation. **Arne M. Malzahn:** Writing – review & editing, Writing – original draft, Methodology, Conceptualization. **Nina S. Liland:** Writing – review & editing, Methodology. **Ikram Belghit:** Writing – review & editing, Writing – original draft, Methodology. **Andreas Hagemann:** Writing – review & editing, Writing – original draft, Visualization, Project administration, Methodology, Funding acquisition, Conceptualization.

## Declaration of competing interest

The authors declare that they have no known competing financial interests or personal relationships that could have appeared to influence the work reported in this paper.
