# Supplementary material for: Retention of prions in the polychaete Hediste diversicolor and black soldier fly, Hermetia illucens, larvae after short-term experimental immersion and feeding with brain homogenate from scrapie infected sheep
Source: Heliyon. 2024 Jul 20;10(15):e34848. doi: 10.1016/j.heliyon.2024.e34848 (PMC11336280; doi:10.1016/j.heliyon.2024.e34848)
Supplement: Multimedia component 1 [file mmc1.docx]

**Supplementary Table 1:** Results from ELISA (HerdChek* Bovine Spongiform Encephalopathy (BSE)-Scrapie Antigen Test Kit from IDEXX) and WB (TeSeE Western Blot from Bio-Rad) analyses for all groups of polychaetes larvae and Black Soldier Fly larvae. *the OD value indicated is the mean of triplicates. Cut off value: 0,141

|  |  | **Elisa**  **OD value*** | **ELISA**  **Results** | **Western Blot** |
| --- | --- | --- | --- | --- |
| **Polychaetes larvae short** | Group 1 | 0,065 | Negative | Negative |
|  | Group 2 | 0,105 | Negative | Negative |
|  | Group 3 | 0,026 | Negative | Negative |
|  | Group 4 | 0,059 | Negative | Negative |
| **Polychaetes larvae Long** | Group 1 | 0,031 | Negative | Negative |
|  | Group 2 | 0,034 | Negative | Negative |
|  | Group 3 | 0,024 | Negative | Negative |
|  | Group 4 | 0,037 | Negative | Negative |
| **BSF larvae short** | Group 1 | 0,175 | **Positive** | **Positive** |
|  | Group 2 | 0,211 | **Positive** | **Positive** |
|  | Group 3 | 0,032 | Negative | Negative |
|  | Group 4 | 0,032 | Negative | Negative |
| **BSF larvae Long** | Group 1 | 0,047 | Negative | Negative |
|  | Group 2 | 0,041 | Negative | Negative |
|  | Group 3 | 0,045 | Negative | Negative |
|  | Group 4 | 0,032 | Negative | Negative |
| **BSF larvae extra long** | Group 2 | 0,055 | Negative | Negative |
|  | Group 2 | 0,044 | Negative | Negative |

**
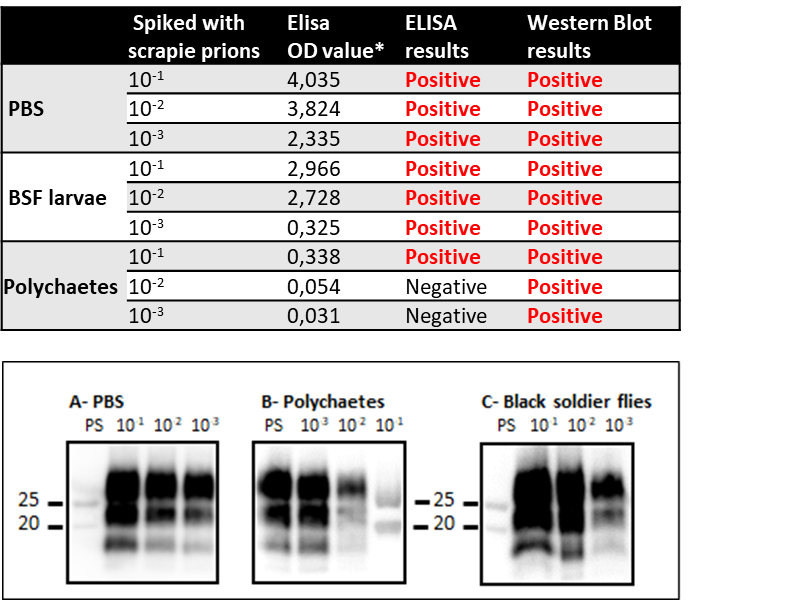
**

**Supplementary Figure 1:** Supplementary Figure 1: Effects of larvae tissue homogenates on the ELISA (HerdChek* Bovine Spongiform Encephalopathy (BSE)-Scrapie Antigen Test Kit from IDEXX) (upper panel) and western blot (TeSeE Western Blot from Bio-Rad) (bottom panel) results. Three different dilutions (10^-1^ 10^-2^ and 10^-3^) of the same 20% scrapie sheep brain homogenate as used for the exposure experiment was mixed 1:1 with PBS or with the worm homogenates. *Cut off value: 0,141; A: PBS (control), B: polychaetes larvae homogenate, C: Black Soldier Fly larvae homogenate; PS: Protein standards. Numbers next to each square indicate molecular weight markers in kDa. The mAb SHa31 was used to visualise PrP^Sc^ after digestion with Proteinase K. The worm tissues, especially the polychaetes tissues, interfered with the test ELISA. Using WB, PrP^Sc^ was equally detected in the larvae homogenates as in PBS. For both tests, no signal was detected in the absence of added scrapie prions (data not shown).


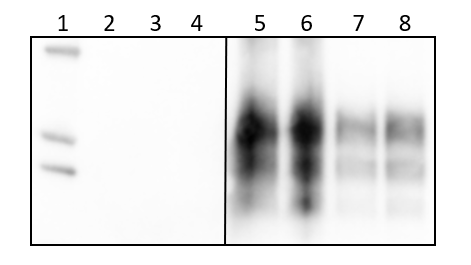


**Supplementary Figure 2:** Representative PMCA controls. PrPSc is visualised by western blot after Proteinase K digestion. The following controls were appropriately included in each WB run: 1: Protein standard, 2: PMCA product without seed (PrPSc), 3: Round 3 PMCA product from homogenate with whole normal BSFL, 4: Round 3 PMCA product from homogenate with whole normal polychaetes larvae, 5: BSFL homogenate spiket 1:20 with scrapie brain homogenate, 6: Polychaetes homogenate with scrapie brain homogenate spiket 1:20 with scrapie brain homogenate, 7: Round 3 PMCA products from scrapie brain 10-2, 8: Scrapie brain homogenate 1:4.
